# Q Fever Osteoarticular Infection in Children

**DOI:** 10.3201/eid2609.191360

**Published:** 2020-09

**Authors:** Halima Dabaja-Younis, Michal Meir, Anat Ilivizki, Daniela Militianu, Mark Eidelman, Imad Kassis, Yael Shachor-Meyouhas

**Affiliations:** Ruth Rappaport Children’s Hospital and Bruce Rappaport Faculty of Medicine, Haifa, Israel

**Keywords:** pediatric, osteomyelitis, osteoarticular, Q fever, *Coxiella burnetii*, vector-borne infections, bacteria, zoonoses

## Abstract

Q fever osteoarticular infection in children is an underestimated disease. We report 3 cases of Q fever osteomyelitis in children and review all cases reported in the literature through March 2018. A high index of suspicion is encouraged in cases of an unusual manifestation, prolonged course, relapsing symptoms, nonresolving or slowly resolving osteomyelitis, culture-negative osteomyelitis, or bone histopathology demonstrating granulomatous changes. Urban residence or lack of direct exposure to animals does not rule out infection. Diagnosis usually requires use of newer diagnostic modalities. Optimal antimicrobial therapy has not been well established; some case-patients may improve spontaneously or during treatment with a β-lactam. The etiology of treatment failure and relapse is not well understood, and tools for follow-up are lacking. Clinicians should be aware of these infections in children to guide optimal treatment, including choice of antimicrobial drugs, duration of therapy, and methods of monitoring response to treatment..

## Abstract 2

Studies of this condition, which is underestimated in children, will aid in its diagnosis and treatment.

Q fever is a zoonotic disease caused by the intracellular bacterium *Coxiella burnetii*. Persistent focalized Q fever infection in adults mainly manifests as endocarditis or as an endovascular infection. Cases of osteoarticular infection (OAI) have been scantly reported in the literature rarely in children (*1*,*2*). Disease severity varies, similar to the clinical variations reported in adult patients (*1*).

*C. burnetii* infections are endemic to Israel. Because diagnosis requires a high level of suspicion, an increase in diagnoses over time may be partly related to physician awareness of the disease rather than true higher incidence (*3*). An observational study of 2,434 cases of *C. burnetii* infection in France (*4*) reported 58 pediatric cases, among which 22 (38%) were OAIs. This large study described the clinical characteristics of Q fever, the less common manifestations of Q fever such as lymphadenitis and lymphoma, and identified risk factors and screening tools predicting complications and death.

Because *C. burnetii* bacteria do not grow in standard laboratory cultures, serology is the first-line diagnostic method for *C. burnetii* infection. Phase II antibodies are predominant during primary infection and Phase I antibodies in persistent infection. Cutoffs of titers considered positive are debated and vary in different countries (*5*,*6*). Immunofluorescence assay (IFA) remains the preferred serology test because of its simplicity and accuracy. Complement fixation test (CFT) is more widely used despite its lower sensitivity (*1*,*2*,*5*). Immunohistochemistry and quantitative PCR of *C. burnetii*–infected tissues are also available (*5*,*6*).

Diagnosis may be aided by clinical criteria. One definite criterion, 2 major criteria, or 1 major and 3 minor criteria are needed for definitive diagnosis of persistent Q fever. Definite criteria include a positive result on culture, PCR, or immunochemistry of bone, synovial biopsy, or joint aspirate. Major criteria include positive blood culture or PCR, phase I IgG antibodies >800, evidence of bone or joint involvement by computed tomography scan, ultrasonography, magnetic resonance imaging (MRI), or abnormal positron emission tomography scan or indium leukocyte scan. Minor criteria include phase I IgG titer of 400–800 mg/dL, temperature >38°C, and mono- or polyarthralgia. These last diagnostic criteria have been proposed to enable diagnosis of *C. burnetii* persistent infection in cases in which titers are below the serologic cutoff (*5*). Other studies use a higher serology cutoff of phase I IgG >1,024 (*6*).

Optimal antimicrobial treatment for chronic Q fever OAI has not been well established. Pediatric treatment recommendations in Q fever OAI are based on treatment of Q fever endocarditis in adults (*7*). We describe 3 cases of Q fever osteomyelitis in children in Israel and a review of the related literature.

## Case 1

**Figure 1:**
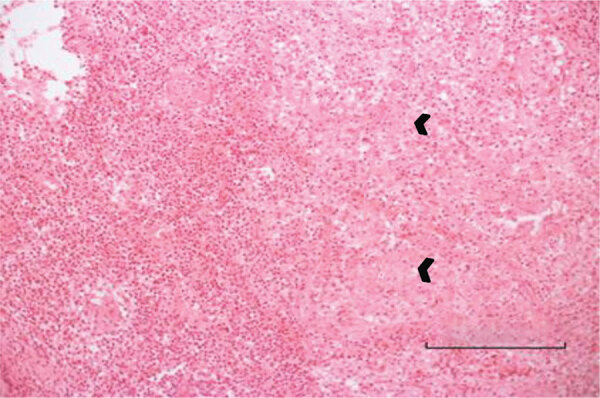
Bone biopsy specimen for a 3-year-old boy (case 1) with Q fever osteoarticular infection, Israel. Hematoxylin and eosin stain shows an acute inflammatory process with neutrophil and lymphocyte predominance. Small arrows indicate giant cells and epithelioid granuloma without necrosis. Bar indicates the diameter of a giant granuloma.

A previously healthy 3-year-old boy was admitted for care with a limp of his right leg and swelling of his right ankle that began 3 weeks before admission. He had no history of trauma and had no fever or other systemic signs of infection. Results of complete blood count (CBC), C-reactive protein (CRP), and radiographic studies at admission were normal. MRI was performed and showed a lytic lesion in the talus bone, suspected to be a malignant space-occupying lesion (Figure 1). Open-bone biopsy was thus performed. Pathology revealed an acute inflammatory process with neutrophil and lymphocyte predominance, giant cells, and an epithelioid granuloma without necrosis, suggesting an infectious process (Figure 1). A swab sample from the tissue was found to be sterile despite the lack of previous antimicrobial therapy. Fungal and mycobacterial PCR results from the paraffin-embedded specimen were negative. Repeated physical examinations revealed signs of cellulitis around the surgical wound with no other systemic manifestations. The patient was treated with a first-generation cephalosporin for 6 weeks, and his clinical signs and symptoms were resolved completely. 

Six months after his discharge, the child experienced swelling and mild cellulitis around his right ankle with no other symptoms. Synovial fluid from the ankle was sampled; bacterial, fungal, and mycobacterial cultures were all negative. The patient then recovered without any treatment. At 1 year after his initial admission, the patient experienced cellulitis at the same site. He was in good general health with no systemic signs of infection. Fluid aspirated from the right ankle, identified as pus, was cultured and analyzed using 16SrRNA PCR. Antimicrobial therapy with a first-generation cephalosporin was reinitiated, with good clinical response. Later in the treatment period, PCR results were found to be positive for *C. burnetii*. IFA confirmed the diagnosis with high titer for phase I IgG, 1:6,400. Transthoracic echocardiography performed 1 month after diagnosis showed no valve involvement.

In light of clinical and radiological evidence of chronic osteomyelitis along with laboratory evidence (positive 16Sr RNA PCR and positive serologic results for *C. burnetii*), the patient was treated for persistent focalized Q fever. Treatment regimen included ciprofloxacin and rifampin for 12 months; full clinical and complete radiological resolution resulted.

Six years after his first infection, after being asymptomatic for 4 years, the patient again experienced pain and tenderness of the contralateral (left) foot and ankle without fever or systemic signs. Results of laboratory studies were unremarkable, but MRI of the ankle showed a Brodie’s abscess of the distal tibia and sonography of the ankle showed a small amount of fluid. Results of testing for 16SrRNA from synovial fluid was negative. IFA revealed high titers for phase I IgG, 1:3,200. On the basis of the patient’s history, clinical signs, and serology, treatment was begun with doxycycline, hydroxychloroquine, and rifampin. Shortly after treatment was started, the patient reported a new elbow pain. MRI study of the right elbow showed synovitis of the elbow and Brodie’s abscess of the distal humerus. Based on the presumed diagnosis of Q fever multifocal recurrent osteomyelitis, triple antibiotic treatment was continued, and substantial improvement was seen by 4 months later. MRI study at the end of treatment revealed complete resolution of all pathological findings. Nearly 12 months after completing his treatment, the patient was asymptomatic. 

## Case 2

**Figure 2:**
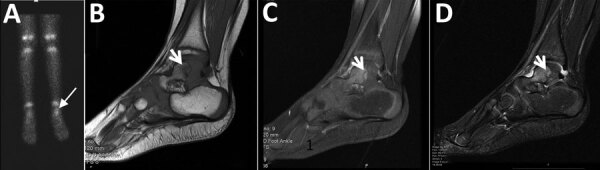
Imaging of the left ankle for a 2-year-old boy (case 2) with Q fever osteoarticular infection, Israel. A) A nuclear bone scan showing uptake in the talus (arrow). B–D) Magnetic resonance imaging sagittal T1 (B), sagittal T1 fat saturation + contrast (C), and sagittal short-TI inversion recovery (D) showing a lesion (white arrows) in the posterior aspect of the talus, noted to be an intramedullary Brodie's abscess in evolution, surrounded by intramedullary edema and accompanied by fluid in the joint.

A previously healthy 2-year-old boy experienced limping for >3 weeks and had a recurrent low-grade fever in the week before admission. He had no history of trauma, exposure to animals, or ingestion of unpasteurized dairy products. At the time of admission, a physical examination noted obvious limping on his left leg but no localized tenderness or focal inflammatory signs. Results of CBC, CRP, and radiographic imaging of his lower limbs were unremarkable. Nuclear imaging (Technetium bone scan) demonstrated an increased signal in the talus of his left ankle (Figure 2, panel A). MRI study of the left ankle showed an intramedullary lesion of the talus compatible with an abscess (Figure 2, panels B, C, D). Empiric treatment with intravenous first-generation cephalosporin was initiated. After 2 weeks of therapy, the patient was only mildly improved. Bone biopsy was not performed because the location of the lesion was unreachable; serology for *C. burnetii* was performed, considering the prolonged symptoms and suboptimal response to therapy. After 6 weeks of antimicrobial therapy, although the patient no longer had symptoms or signs of infection, the *C. burnetii* serology result was unexpectedly positive: phase I IFA IgG titer was 1:200. 

Confirmatory serology performed 2 weeks later showed a phase I IFA IgG titer of 1:800. At that time, the patient remained asymptomatic. A repeated MRI of the ankle showed the same lesion with no major changes. Results of a transthoracic echocardiogram, performed 3 months after initial care because of concern for possible endocarditis, were unremarkable. Considering the chronic course of Q fever OAI and the risk for relapse or progression to other manifestations, combined therapy of rifampin and trimethoprim/sulfamethoxazole (TMP/SMX) was initiated. The patient completed 1 year of treatment, throughout which he continued to be asymptomatic. MRI 12 months after treatment showed complete resolution of the primary talar lesion. Phase I IFA IgG titer at the end of treatment was 1:200.

## Case 3

**Figure 3:**
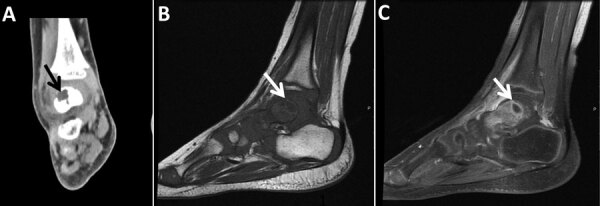
Imaging of the left ankle for a 3-year-old boy (case 3) with Q fever osteoarticular infection, Israel. A) Computed tomography imaging, coronal view, shows a lytic lesion in the talus (black arrow). B, C) Magnetic resonance imaging sagittal T1 (B) and sagittal T1 fat saturation + contrast (C) demonstrate a lesion in the posterior aspect of the talus (white arrows), determined to be an intramedullary abscess (Brodie’s abscess) surrounded by edema.

A previously healthy 3-year-old boy experienced swelling and tenderness over his left foot and calcaneus for 6 months before his admission. His body temperature was normal over that period, and he had no systemic signs of infection. Two falls and mild bruises of the same leg were reported around the time his symptoms began. The patient lived in a rural area and was exposed to livestock. Results of CBC, CRP, erythrocyte sedimentation rate (ESR), and radiographic studies conducted at the time of admission were unremarkable. MRI demonstrated synovitis of the small joints of the midfoot (Figure 3). The patient underwent fine-needle aspiration of the ankle, which was technically difficult. Synovial fluid from this aspiration was sterile despite no previous antimicrobial therapy; results of 16SrRNA and specific PCR for *C. burnetii* were negative.

Serology for Q fever was positive with high titer of phase I IgG, 1:1600, and of phase II, 1:1600, in IFA. Transthoracic echocardiography performed 1 month later showed no cardiac valve involvement. On the basis of the suggestive clinical signs, potential exposure to farm animals, and positive serology for *C. burnetii*, the patient was treated with rifampin and TMP/SMX for Q fever OAI. Repeated serology showed increasing titers: IgG phase II up to 6,400, and IgG phase I up to 3,200. Despite 8 months of antimicrobial treatment, the patient remained symptomatic with debilitating pain. MRI performed at that time revealed synovitis and osteomyelitis with intramedullary abscesses in the small bones of the midfoot, not amenable for drainage. Twelve months into his antimicrobial therapy, his health had improved overall, with decreasing pain and decreasing IgG phase I titers.

## Literature Review

A review of the literature revealed 8 articles describing 29 pediatric cases of OAI caused by *C. burnetii* (*9**–**16*) (Appendix Table). One of the 3 cases we describe was also described briefly in a published case series (*16*). Of the 31 total patients described, 24 (77.41%) were male; 29 (93.54%) were <10 years of age. All were previously healthy except for 1 patient who had acute lymphoblastic leukemia. Exposure to livestock or household animals was reported in most cases. Only 5 of 10 patients in Israel, including 2 of our 3 case-patients, led urban lifestyles (*9**–**16*).

Clinical manifestations were subtle or occult for 20 (65%) of patients. In cases in which laboratory tests were reported, CBC and CRP results were mostly within reference ranges. ESR values, considered superior to other acute-phase reactants for assessment of chronic infections, were not reported for most cases of Q fever OAI.

In cases in which biopsies were performed, histology was consistent with inflammation and noncaseating granulomas. Results of bone or synovial biopsy and joint aspirate cultures were always negative; results for 16-S rRNA gene PCR for *C. burnetii* were positive in most of the joint aspirate cultures. In 16 (52%) cases, bone lesions were surgically debrided. 

Most patients received combined therapy for 6–36 months. One case-patient was treated for a limited duration of 6 weeks with good initial response, but no data regarding long-term recovery were available. Three patients received no antimicrobial therapy, but they all underwent surgical debridement. Evidence of relapse was noted in 1 of those patients; data were unavailable for the other 2 patients because the follow-up period for those was brief. 

In 18/31 cases (58%), the disease was multifocal, and in most cases a relapsing-remitting course was observed regardless of appropriate antimicrobial treatment. Eleven (35%) of 31 children had >1 recurrence; others had no reported recurrence. The observation of no recurrence may be biased; some cases were published shortly after completion of the patients’ course of antimicrobial treatment, which precluded a substantial follow-up period (*9**–**16*). In cases in which serology was monitored during and after treatment, serology remained unchanged or even increased during follow-up, which can be explained by the unreliable role of serology as a test of cure in this disease (*9**–**16*).

## Discussion 

We describe 3 cases of definite *C. burnetii* OAI in children and provide a review of the literature for these cases. Diagnosis for these 3 patients was defined by the prolonged clinical symptoms associated with microbiological evidence. Major criteria including clinical evidence of OAI, bone or joint involvement visible on MRI, and diagnostic serologic titer were found in all 3 cases. The first case also had a positive *C. burnetii* PCR as a definite criterion (*5*,*8*). 

Our review of the published cases with chronic Q fever OAI sheds additional light on this often underdiagnosed and easily missed infectious disease. A high index of suspicion is encouraged in cases of an unusual case manifestation, a chronic or subacute course, relapsing symptoms and signs, nonresolving or slowly resolving osteomyelitis, culture-negative osteomyelitis, exposure history to farm animals, or bone histology demonstrating granulomatous changes (*7*). Some cases improve spontaneously or during treatment with a β-lactam, which is not active against *C. burnetii*, and have no obvious rural contact, making diagnosis more challenging. Optimal antimicrobial therapy for chronic Q fever OAI has not been well established.

Current treatment recommendations are based on case series, retrospective cohort studies, and in vitro data. In the 3 cases we describe, the choice of treatment was based on recent literature and the safety and availability of the suggested drugs for the specific patient. The patient in case 1 was treated with ciprofloxacin and rifampin. Doxycycline therapy is considered ill-advised in children <8 years of age because of its side effects of teeth staining and weakening of enamel, especially in prolonged treatment. Of note, the US Centers for Disease Control and Prevention (CDC) in 2013 recommended the of use of doxycycline at a dose of 2.2 mg/kg twice per day for 2 weeks in children <8 years of age for the treatment of acute and chronic Q fever in United States (*17*). However, Q fever OAI requires a much longer treatment period with doxycycline, with a higher likelihood and potential for long-term adverse effects. Doxycycline is not approved in Israel for chronic use in children <8 years of age. Hydroxycholoroquine was not available in a liquid form and required special pharmacy preparation which could interfere with treatment continuity.

We diagnosed OAI in the other 2 case-patients shortly after the recurrence of osteomyelitis in case-patient 1. Considering the recurrence occurred while this patient was under treatment with ciprofloxacin and rifampin, we considered treatment failure using this regimen and therefore chose rifampin and TMP/SMX for the other 2 patients. TMP/SMX has been used in combination with doxycycline, ciprofloxacin, or rifampin according to reports available at that time (*11*,*12*).

The standard treatment of Q fever–persistent focalized infection in adult patients is hydroxychloroquine and doxycycline; this regimen leads to fewer recurrences. Minimal treatment duration in persistent infections is 18 months; we have no evidence to determine the duration of doxycycline and hydroxychloroquine therapy (*8*,*18*,*19*). The effectiveness of this therapy is likely to result from the alkalizing effect of chloroquine and its derivative, the hydroxyl, on lysosomal compartments, thus enabling improved doxycycline activity (*20*).

For children <8 years old, treatment with TMP/SMX or rifampin is preferred because of the risk for dental staining with prolonged tetracycline therapy (*7*). In vitro studies showed complete susceptibility to rifampin, TMP/SMX, and tetracyclines; heterogenous susceptibility to fluoroquinolones and erythromycin; and a little inhibitory growth effect of β-lactam antimicrobial drugs (*21*). Studies show no consensus regarding optimal treatment duration of Q fever OAI in children. Minimum treatment duration was 6 months in most of cases reviewed. Appropriate drug therapy did not necessarily prevent recurrences; in 16 (53%) cases, patients required surgical intervention despite adequate antimicrobial therapy (*9**–**16*). In addition to antimicrobial and surgical treatment, immunomodulatory agents including interferon gamma were found to be effective in vitro and in some case reports of chronic Q fever (*10*,*22*).

The etiology of treatment failure and relapse in Q fever OAI is not well understood. We were not able to conclude which treatment regimen was associated with the lowest recurrence rate; the same treatment regimens led to cure in some cases and treatment failure or relapse in others. We observed clinical recurrence and increasing antibody titers despite extended combined antimicrobial drug therapy (*23*).

Whether recurrence is related to reduced drug levels is unknown. In most cases, drug levels in blood or hair samples was not considered. In our institution, these tests were not routinely available. Doxycycline hair level assay testing to determine long-term compliance was deemed unnecessary because most of our patients were too young for treatment with doxycycline. No documentation of compliance was described; however, in our 3 cases, parents reported orange-stained urine during follow-up visits, indicating good compliance for rifampin. Our physicians had also evaluated parents administering medications as caring, dedicated, and reliable. Another factor that may explain the nonnegligible rate of recurrence is the dormancy of the intracellular bacterium. These dormant infectious particles turn to metabolically active in response to acidification of the endosome and are associated with the development of the parasitephorous vacuoles that allow the organism to replicate repeatedly. (*24*,*25*) 

Tools for follow-up are lacking. Acute-phase reactants were absent or only mildly elevated in in 17/20 (85%) cases, in which laboratory data were documented. Radiographic resolution is not expected over a period of several weeks. Serology may not change throughout the first years following clinical cure (*2*,*5*,*7*). We did not user newer imaging tools such as the 18F FDG-PET/CT described in recent studies (*4*,*5*,*26*) for follow-up in our cases.

In summary, pediatric OAI with prolonged course or inadequate response to empiric treatment should raise the suspicion of unusual pathogens such as *C. burnetii*. Further studies are needed to guide optimal treatment of Q fever OAI, including choice of antimicrobial drugs, duration of therapy, and methods of monitoring response to treatment. Diagnosis of Q fever OAI requires increased awareness and use of newer diagnostic modalities, and urban residence or lack of direct exposure to animals does not rule out infection, especially in countries in which Q fever is endemic. 

Appendix Additional information about Q fever osteoarticular infection in children.
